# Micro RNAs are involved in activation of epicardium during zebrafish heart regeneration

**DOI:** 10.1038/s41420-018-0041-x

**Published:** 2018-03-12

**Authors:** Marcello Ceci, Claudia Carlantoni, Maria Azzurra Missinato, Davide Bonvissuto, Bruna Di Giacomo, Riccardo Contu, Nicla Romano

**Affiliations:** 1 Department of Ecological and Biological Sciences, University of Tuscia, Viterbo, Italy; 2 Department of Developmental Genetics, MPI-Heart and Lung Research, Bad Nauheim, Germany; 3 Department of Developmental Biology, University of Pittsburgh School of Medicine, Pittsburgh, PA USA; 4 Faculty of Medicine and Surgery, University of Sacred Heart, Rome, Italy; 5 Department of Medicine and Research Service, Cardiology Section, Veterans Administration San Diego Healthcare System, University of S.Diego, San Diego, CA USA

## Abstract

Zebrafish could be an interesting translational model to understand and improve the post-infarction trial and possible regeneration in humans. The adult zebrafish is able to regenerate efficiently after resecting nearly 20% of the ventricular apex. This process requires the concert activation of the epicardium and endocardium, as well as trans-differentiation of pre-existing cardiomyocytes that together replace the lost tissue. The molecular mechanisms involved in this activation process are not completely clarified. In this work, in order to investigate if the downregulation of these miRNAs (miRs) are linked with the activation of epicardium, the expressions of miR-133a, b and miR-1 during regeneration were analysed. qPCR analyses in whole-heart, or from distinct dissected epicardial cells comparing to regenerative clot (containing cardiomyocytes, fibroblasts and endocardial cells) by a laser-micro-dissector, have indicated that already at 24 h there is a downregulation of miRs: (1) miR-133a and miR-1 in the epicardium and (2) miR-133b and miR-1 in the regenerative clot. All the miRs remain downregulated until 7 days post-surgery. With the aim to visualize the activations of heart component in combination with miRs, we developed immunohistochemistry using antibodies directed against common markers in mammals as well as zebrafish: Wilms tumour 1 (WT1), a marker of epicardium; heat-shock protein 70 (HSP70), a chaperon activated during regeneration; and the Cardiac Troponin T (cTnT), a marker of differentiated cardiomyocytes. All these markers are directly or indirectly linked to the investigated miRs. WT1 and HSP70 strongly marked the regeneration site just at 2–3 days postventricular resection. In coherence, cTnT intensively marked the regenerative portion from 7 days onwards. miRs-1 and -133 (a,b) have been strongly involved in the activation of epicardium and regenerative clot during the regeneration process in zebrafish. This study can be a useful translational model to understand the early epicardial activation in which miRs-133a and miR-1 seem to play a central role as observed in the human heart.

## Introduction

The regenerative capacity in the mammalian heart after tissue damage, such as infarction, seems to be limited by replacing dead cardiomyocytes (CM) largely by fibroblasts, because CMs differentiated from resident stem cells are not enough to replace the lost tissue^1,2^. In contrast, natural cardiac regeneration after injury appears to be excellent in lower vertebrates such as fishes or amphibians, and partially conserved among neonatal mammals^3^. In zebrafish, epicardial activation and initiation of myocardial proliferation are able to efficiently regenerate through after the resection of 20% of the ventricular apex of the heart^4^. The cardiac environment created by CMs and non-muscle cells after injury is believed to be critical in facilitating the regenerative response^5^. Regeneration after amputation of the ventricular apex has occurred with the same sequence of events as cardiac cryoinjury. This latter procedure was recently suggested as an alternative technique to reproduce the infarction event in zebrafish^6^. During the regenerative process, the epicardium plays a primary role^1,5,7^ due to its derivation: a progenitor pool derives from the mesodermal coeloma and the neural crest cells^8^. The progenitor pool contributes to build the coronaries and interstitium of heart^9^. The epicardium-derived cells (EDC) and the consequent epicardial cells (EPCs) are essential regulators of cardiac growth and differentiation^10^. EDCs and EPCs respond to fibroblast growth factors (FGFs) in both embryogenesis and regeneration processes and undergo a number of cellular modifications^11^ that is  required to activate the transition from epithelial to mesenchymal cells, such as cytoskeletal re-arrangement and expression of hyaluronan-mediated motility receptor, neccessaries to move in the damage site^ 12,13^. In fact, after the FGF-mediated-activation, EPCs start to migrate into the injury site and promote both neovascularization and myocardial differentiation^13,14^. The microRNAs (miRs) play a regulatory role in the development and homoeostasis of different tissues^15–17^, including the heart^15,17–19^. In the latter, they are involved in the activation of fibroblasts in producing FGFs^20,21^ as well as the hypertrophic response of epithelial and muscular cells after injury to compensate for the loss of contractile tissue^17^. miR-1/miR-133 are mainly implicated in post lesion in mammals as well as in zebrafish^14,17,22,23^. Particularly, miR-133 has two isoforms, miR-133a and miR-133b, and their activity seems to be similar at the moment^23^. The miR-133 expression is regulated by extracellular signal-regulated kinase 1/2 activation and is inversely correlated with vascular growth^23^, since it is strongly related to FGF-receptor expression^24^. In zebrafish, miR-133 antagonism that occurred during FGF-receptor inhibition has accelerated the regeneration of appendage or heart damage through increased proliferation within the regeneration blastema^25^. At 7 days after amputation (dpa), the level of miR-133 expression in the ventricle of the heart was lower than control individuals and suggested that miR-133 is an endogenous inhibitor of EC proliferation^25^. In the rationale, we have suspected that during regeneration, the downregulation of miRs-133a, 133b and miR-1 could occur before 7 days after the resection and that could be differently regulated by the cell-type (i.e., epicardium and CM). To pursue this possibility, we have planned experiments of heart ventricle apex amputation–regeneration in zebrafish focusing on the epicardial activation earlier to 7 dpa. The 20% of ventricle’s apex was resected in adult heart and consequently harvested during regeneration from 1 to 30 dpa, and then investigated the expression of miRs-1 and 133 (a,b) in the explanted heart. Besides miRs expression analysis, we also performed a comprehensive study of the histology and immunohistochemistry related to the epicardium and CM differentiation. The immunohistochemistry has been focused on three markers linked with the investigated miRs: (1) HSP70, as a marker of recovery activities as well as the block of apoptosis and controlled by miRs expression; (2) Wilms tumour 1 (WT1), as a specific marker for development/regeneration activity of the epicardium^26,27^ and in the epithelial–mesenchymal transition (EMT)^28^; (3) cTnT or component of cardiac troponin T, as a marker of differentiated myocardial cells because its expression is essential for sarcomere assembly and directly mediated by miR-1 upregulation^29^. The expected results could demonstrate an early decrease in the expression levels of miRs accompanied with high expressions of HSP70 and WT1, and a different temporal/spatial mode during the regenerative process. In addition, we expect that the miRs downregulation is correlated to the opposite behaviour of the markers: HSP70/WT1 in the epicardium and HSP70/cTnT in the differentiating CMs.

## Results

### Downregulation of microRNAs expression occur already from 1 dpa

**Fig. 1:**
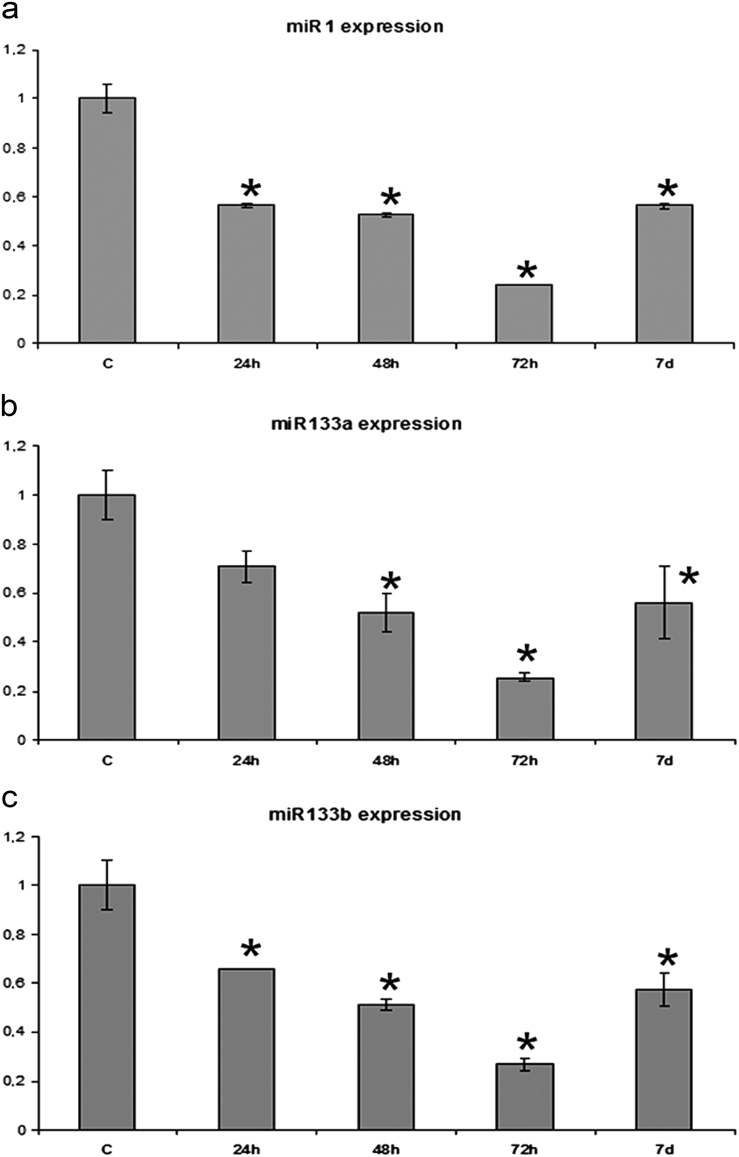
**Analysis of qRT-PCR regarding the relative expression of miR in the heart during the regeneration process at 1, 2, 3 and 7 dpa.** **a** Expression of miR-1; **b** expression of miR-133a; **c** expression of miR-133b. (*) Statistically significant difference in the expression level in comparison to control (*P* < 0.001) according to the Student's *t*-test to a queue. Normalized values with U6 snRNA

**Fig. 2:**
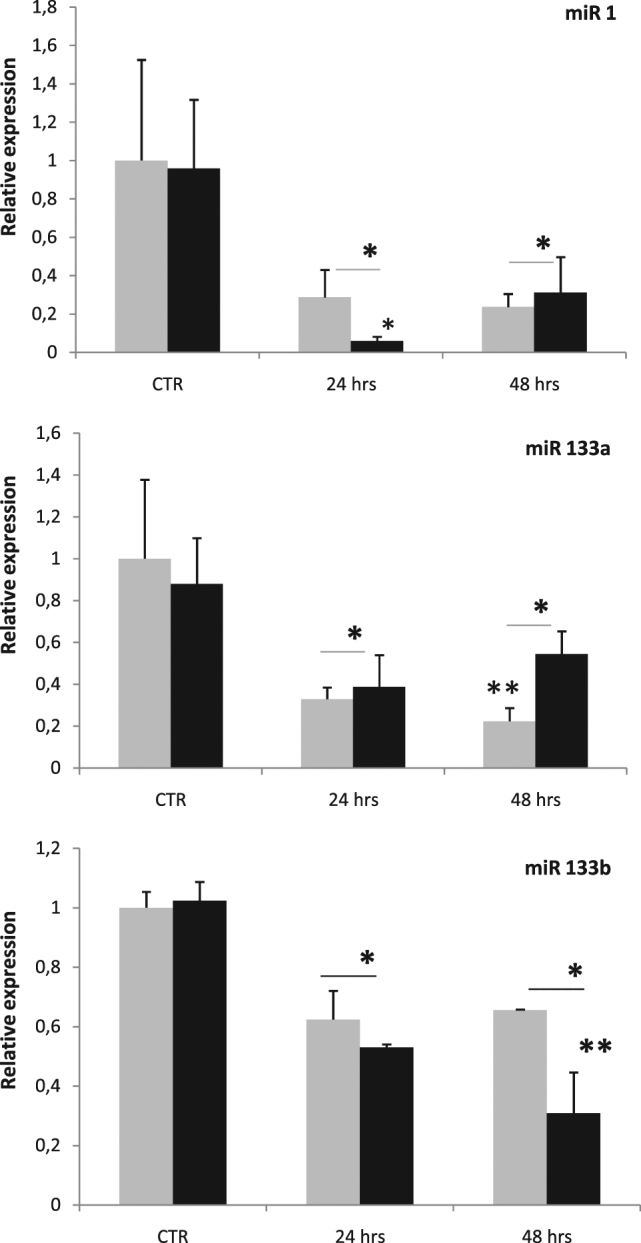
**Quantitative PCR regarding the relative expression of miRs in the epicardial cells (EPCs, light grey) and in regenerative clot (RC, black) of adult zebrafish at 1 (24 h) and 2 (48 h) dpa.** (*) Statistically significant difference in the expression level in comparison to control (*P* < 0.001) according to the Student's *t*-test to a queue. Normalized values with U6 snRNA. (**) Values statistically significant (*P* < 0.001) EPCs vs RC

The analysis by qRT-PCR of the whole-heart of zebrafish in regeneration was carried out at 1, 2, 3, 7 and 30 dpa samples with their respective controls (Fig. 1). All the control values were normalized to 1. The levels of expression of various miRs during heart regeneration have shown a significant decrease compared to the non-amputated control (Fig. 1). In particular, miR-1 and miR-133b have undergone a significant downregulation at 1 dpa. miR-1 at 1 dpa has evidenced a value of 0.566 ± 0.008 and 0.526 ± 0.004 on the second day. At 3 days, the qRT-PCR has shown a decrease of 75% (0.240 ± 0.003) (Fig. 1a). From 7 dpa, the miR-1 expression has been slowly returned to the control values (data not shown). miR-133b (Fig. 1c) decreases by about 42% at 1 dpa (0.657 ± 0.00). At 2 dpa, the expression level is 50% and at 3 dpa, about 75% less than the control (0.513 ± 0.0246 and 0.026 ± 0.0266, respectively) (Fig. 1c). Even at 7 days, the expression of miR-133b is less than approximately 50% when compared to the control (0.574 ± 0.068) and it starts to grow until the regenertion is complete. Regarding miR-133a (Fig. 1b), the qPCR data show that at 24 hpa there is a decrease (but not statistically significant, 0.707 ± 0.065), while the values of expression on days 2, 3 and 7 are similar to those of the miR-1 (0.519 ± 0.079, 0.255 ± 0.016, 0.560 ± 0.145, respectively) (Fig. 2).

The qPCR data on the whole-heart gave intriguing indications of miRs early involvement in the regenerative process. Thus we planned further experiments by using the laser dissection methods to separate EPCs (see supplementary material) from the internal regenerative clot (RC = with endocardial cells, CMs and possibly the contribution of dedifferentiated EPCs). With this aim, and in view to manage a small quantity of RNAs, we decide to use a more sensitive procedure to detect the miRs (taqMan-microRNA assay; Thermo Fisher, US). Laser dissection has allowed to crop and select the EPCs surrounding the RC and also to cut out the clot in a different sample, and analyse the critical period of 24 and 48 hpa in order to investigate the miRs expression in the two portions (supplementary material). The results (Fig. 2) have confirmed the general downregulation of miRs already from 24 hpa. The results indicated that miR-1 has been strongly downregulated in the RC already at 24 hpa (0.060 ± 0.021, *P* < 0.001) as compared to control and EPCs. miR-133a has significantly downregulated at 24 hpa in both portions (EPCs, 0.329 ± 0.056; RC, 0.388 ± 0.152; *P* < 0,001) vs control (EPCs, 1 ± 0.377; RC 0.880 ± 0.219). At 48 hpa, the EPCs has showed a significant downregulation (0.224 ± 0.063; *P* < 0.001) as compared to RC and parallel control samples (0.545 ± 0.108). Also miR-133b has downregulated significantly already at 24 hpa (EPCs, 0.62 ± 0,096; RC 0.530 ± 0.010; *P* < 0.001) as compared to controls (EPCs, 1 ± 0.535; RC, 1.024 ± 0.062). Interestingly, at 48 hpa the RC portion has shown a strong downregulation as compared to EPCs (0.309 ± 0.137; 0.655 ± 0.002, respectively; *P* < 0.001) and control samples.

### Histology during cardiac regeneration

**Fig. 3:**
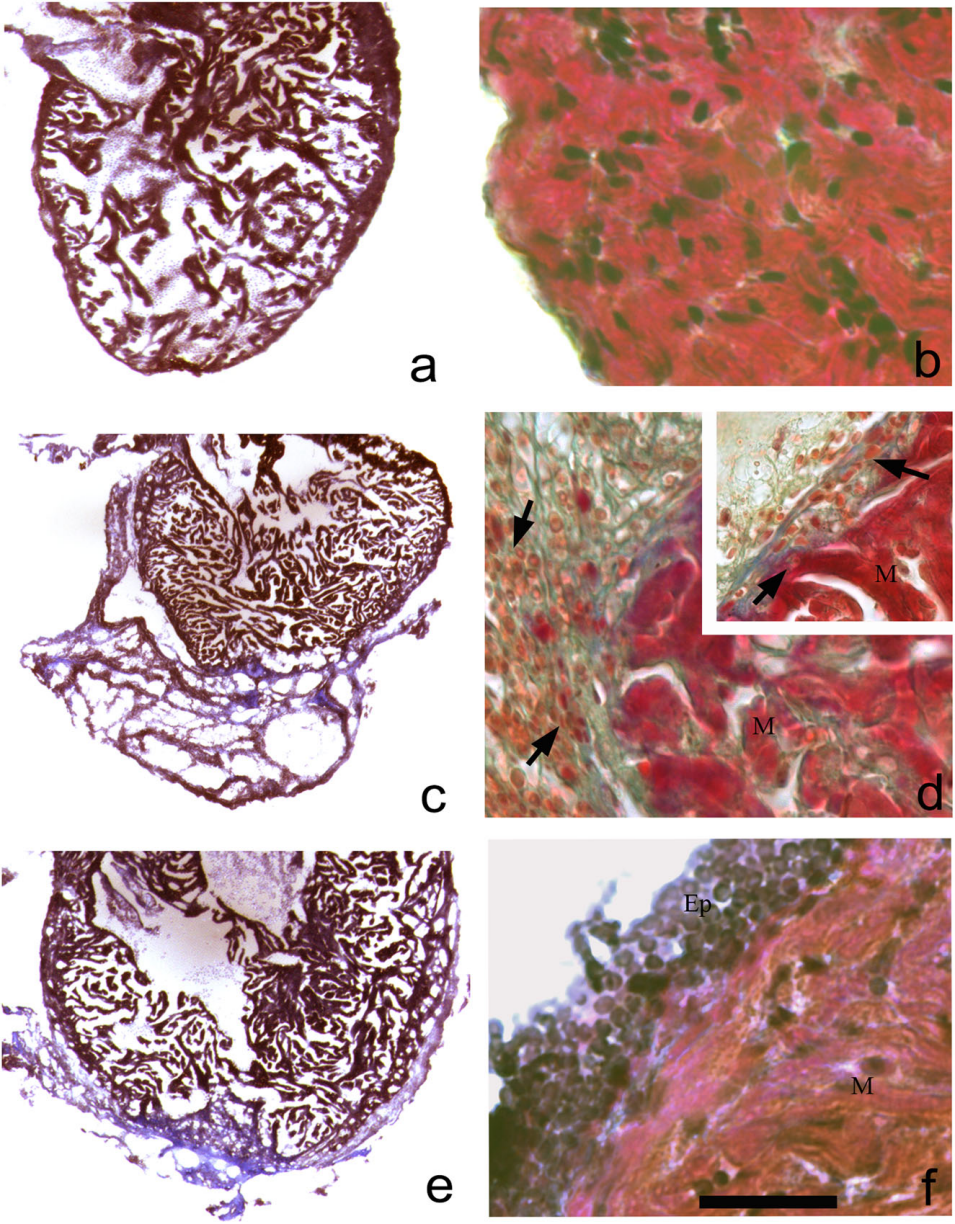
Histological staining using Masson’s trichrome on heart controls (**a**, **b**) and during regeneration (**c–f**). At 2 dpa (**c**) wide regenerative clot is clearly evidenced; in **d** a fibrous clot in blue is observed, while some undifferentiated cells that infiltrated the clot-surrounded epicardium are reactive (**d**, inset). We also notice some macrophages (**d**, inset). A 3 days (**e**) the clot is in resorption; in a magnification (**f**) epicardial stratification is observed. M myocytes, Ep epicardium (**a** bar = 1.5 mm; **b** bar = 45 μm; **c**, **e** bar = 1.5 mm; **d**, **f** bar = 45 μm)

Histology has been performed on samples in regeneration and non-amputated controls (at stages 2, 3, 7, 14 and 30 days post-surgery) and has been stained by Masson’s protocol (Fig. 3). The regions not affected by the amputation have shown a normal colouring comparable to that of the non-amputated samples. Particularly evident from 2–3 post operation days, the staining has revealed a heterogeneity among the cellular elements of the portion interested by resection: (1) an external monolayer of flat cells (EPCs) that has been coming from the epicardium; (2) CMs containing muscular-fibres organization have been intensely stained in red; (3) small cells (average diameter, 4.5 ± 0.5 µm) in the RC has been seemed of undifferentiated-type and stained in blue colour. The portion of the epicardium distant from the RC has been not seemed to be reactive. Among EPCs, has been observed macrophages and fibroblasts. Beside the many fibres of the extracellular matrix and in addition to the RC, also myeloid elements mainly consisting of erythrocyte have been evidenced. From 3 dpa, the clot has begun to be reabsorbed and it has shown some CMs provided of muscular fibres. Contemporarily, the epicardium that surrounded the clot has consisted in 2–3 lines of elements.

### Immunohistochemistry of heart during regeneration confirms the epicardial activation already between 24–48 h post-surgery

#### HSP70

**Fig. 4:**
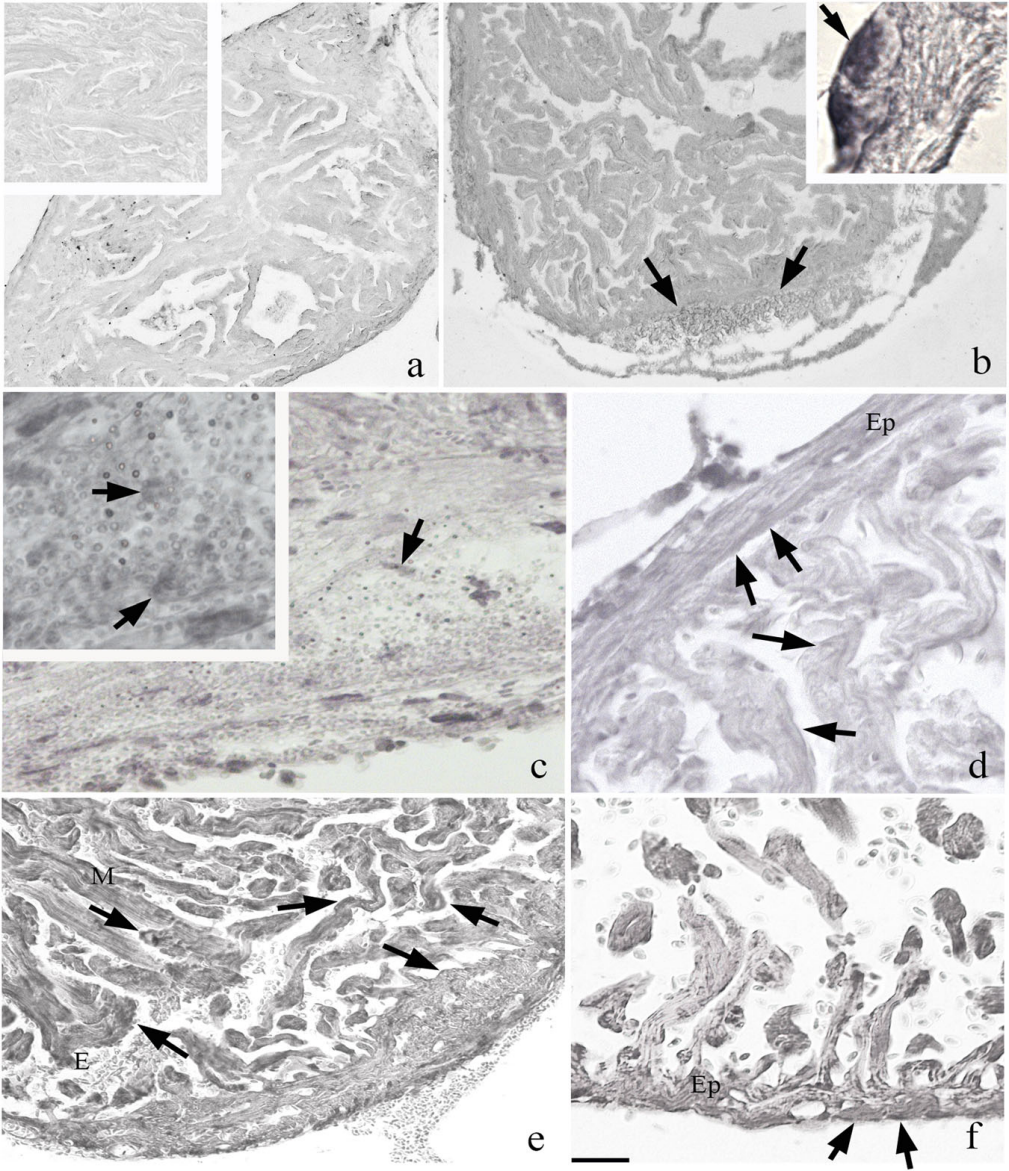
**Immunohistochemistry with HSP70 on zebrafish heart.** **a** HSP70 staining in the control. The reaction is marked in the samples of 2 and 3 dpa (**b** and **d**, respectively); in particular in the regions of the clot (**c**, and inset) and epicardium (Ep) (**b**, inset). In the region of cutting are visible at 2 dpa many rounded positive cells. From 14 days (**f**) onwards, the clot has almost disappeared and replaced by new myocytes (M); is visible to the evidenced several erythrocytes in the heart lumen (**e**). At days 30, (**f**) positivity is still reactive than in the control (**a** bar = 300 μm; inset = 75 μm; **b** bar = 300 μm and inset bar = 4 μm; **c** bar = 75 μm and inset bar = 45 μm; **d** bar = 150 μm and bar = 150 μm; **f** bar = 75 μm)

Immunohistochemistry performed on samples collected from 1–30 dpa has been shown in Fig. 4. In the first 3 dpa, HSP70-positive cardiac cells have been strongly marked in the cytoplasmic region than in the controls and after 7 days onwards, the intensity of reaction has decreased until they reach the control features (not shown). The positivity has been particularly evident in the clot (from 2–7 dpa) and in the epicardium that surrounds it. Inside, the clotsmall-HSP70-positive cells with rounded morphology and without internal myofibrillar organization were observed. The presence of erythrocytes has been predominant with respect to other blood cells (macrophages and a few granulocytes) in the clot. The CMs of the regenerating hearts looked morphologically normal in non-amputated portions.

#### WT1

**Fig. 5:**
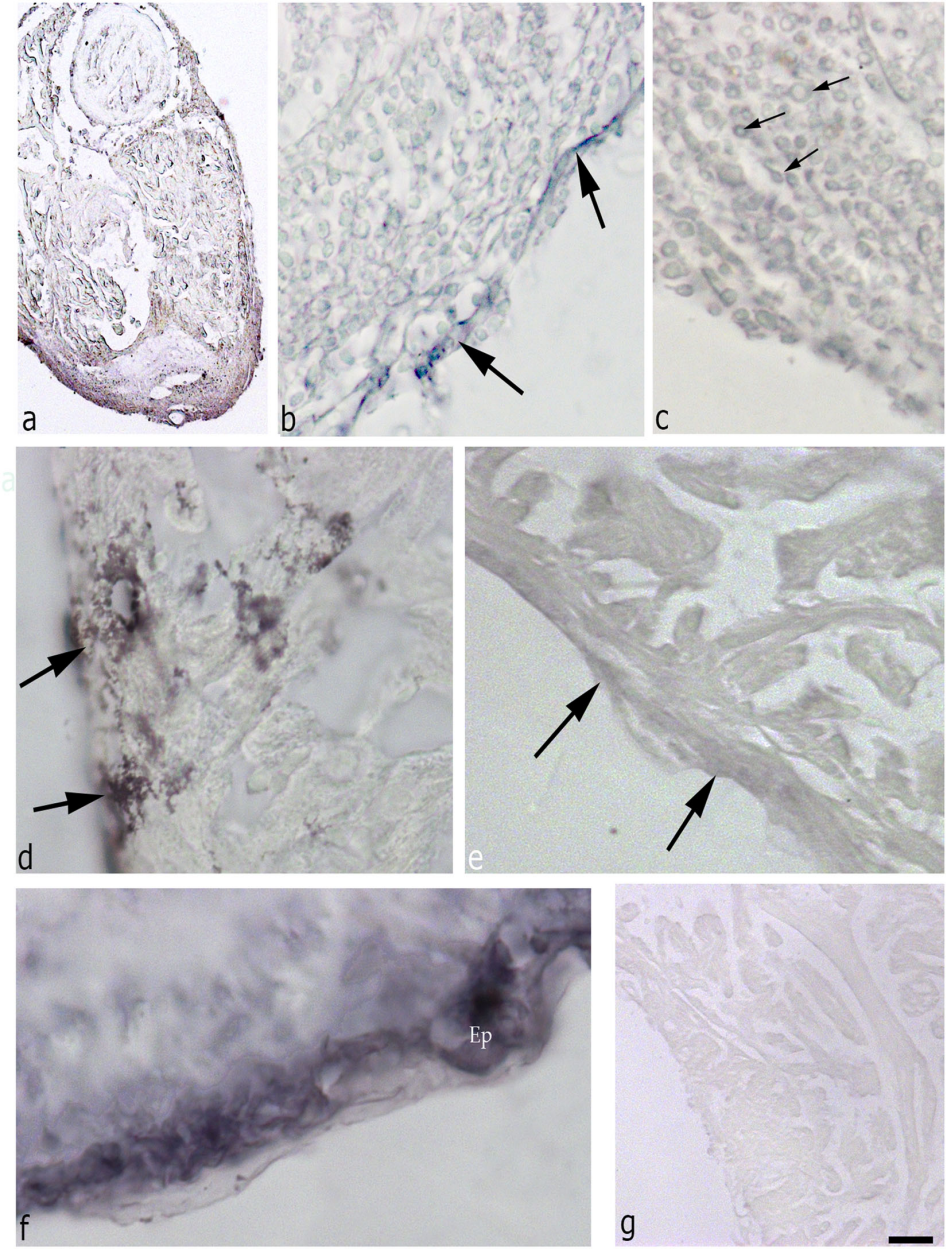
Immunohistochemistry with WT1 on regenerative heart (**a** bar = 300 µm; **b** bar = 150 µm; **c** bar = 75 µm; **d** bar = 45 µm; **e** bar = 50 µm; **f** bar = 45 µm; **g** bar = 150 µm). At 2 days, (**a**) there is a strong reactivity in the epicardium surrounding the organ. This positivity is present also in EPCs surrounding the clot (**b**) and in cells inside the clot (**c**). At 3 dpa, (**d**) the epicardium is reactive, but at 7 dpa (**e**) the signal in some epicardial portions is lower as compared with the parts next to the RC (**f**). In **g**, the control staining (bar = 75 µm). Ep epicardium

The immunohistochemistry with WT1 has been performed to highlight the activation of EPCs because it is considered to be a specific marker of the epithelium during embryogenesis. In all regenerative samples, it was observed that immunohistochemical reaction circumscribed the epicardium (Fig. 5). In the control, the reaction was very slight, as well as the hearts in regeneration at day 30. In contrast, at 1 dpa, a strong marking of the epicardium at the level of whole-heart was observed. In particular, in the ventricle, the positivity in the day 2 dpa has been evident in all EPCs and even inside the clot (limited to small rounded cells). At day 3 dpa, the positive signal is higher in the epicardium and mostly in EPCs surrounding the clot. At later time points the reactivity gradually diminished, up to 30 days post amputation, when the antibody-positivity has been comparable to that of the non-amputated control (data not shown).

#### cTnT

**Fig. 6:**
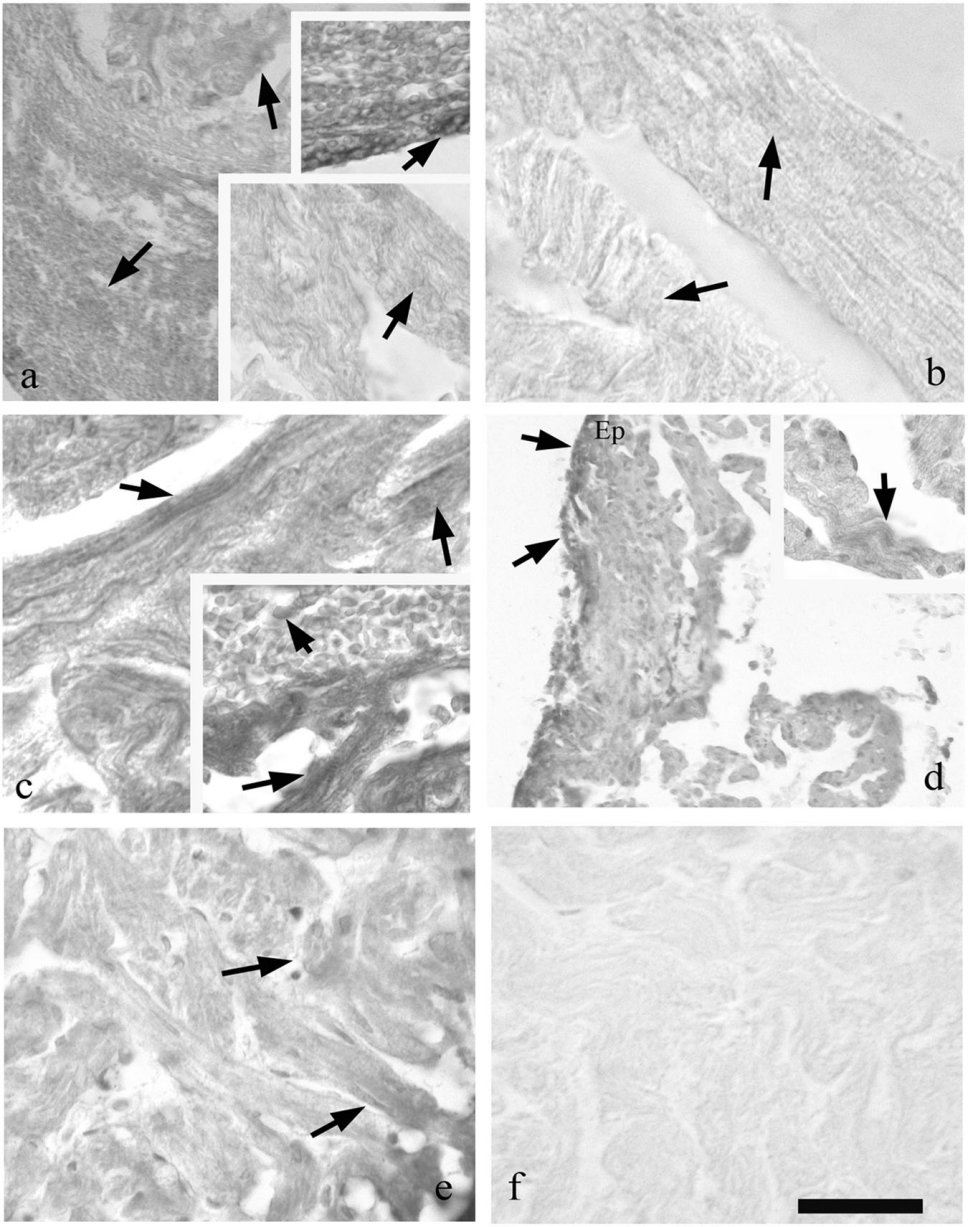
Immunohistochemistry with cTnT on regenerating heart (**a**, **e** lower bar = 75 μm and upper inset bar = 45 μm; **b** bar = 54 μm; **c** bar = 45 μm and inset bar = 54 μm; **d**, bar = 150 μm and inset bar = 75 μm; and bar = 54 μm). At 2 days (**a**) are observed healthy muscle fibres with point reactivity (inset bottom) and highly reactive rounded cells (upper inset) among the heatly fibres. At 3 (**b**) and 7 (**c**) dpa next to healthy fibres (M) cTnT^+^ cells are evidenced close the clot (**c**, inset). As is shown in 14 days, (**d**) the epicardium (Ep) is cTnT posititve; infiltrated cells in the clot are also positive. At 30 dpa, (**e**) muscle fibres are reformed in the region of the cut and the reactivity for cTnT is comparable to the control. In **f** is the sample stained with omission of cTnT that shows no reactivity

Immunohistochemistry performed with cTnT antibody showed a reactivity point on muscle fibres normal and comparable to control in all healthy portions of the myocardium itself (Fig. 6). In the portions of the myocardium facing the area of the surgical cut/postoperative clot, the reaction has been appeared to be more intense. This has been particularly detectable in healthy fibres in all regenerative trial from 7 dpa and close to the clot. Inside the RC, sparse undifferentiated rounded cells were apparently positive to the T-troponin already at 3 dpa.

## Discussion

**Fig. 7:**
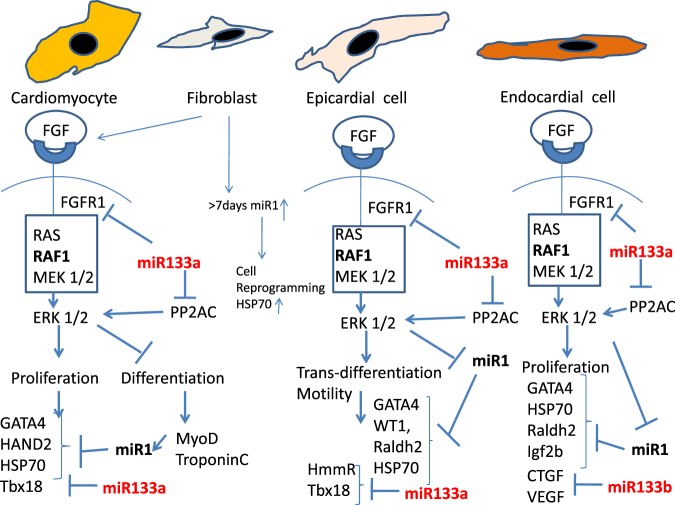
**A proposed schematic model of miR-1 and miR-133 actions in blocking the FGF-dependent transduction pathway in the cells involved in cardiac regeneration: CMs, fibroblast, EPCs and endocardial cells.** In zebrafish these four types acting in concert, the regeneration in trans-differentiation and/or proliferation of existing resident cells under the induction of FGF1-2. The scheme is a comprehensive information from literature and from the present research. miR-1 is not expressed by ERK1,2 activity because of the expression of cardiac embryonal genes and relative proteins such as GATA4. When the genes of differentiation are expressed, such as MyoD, miR-1 start to be highly transcripted and act as a repressor of GATA4 translation and other embryonic key proteins. For example, in epicardial cells it may control the WT1 expression. miR-133a instead acts directly on blocking the expression of the receptor of FGF and on the expression of the PP2AC that promotes the activity of ERK. miR-133b is instead involved in the transition from endothelial to mesenchymal cells by blocking directly the expression of connective tissue growth factor (CTGF)

Since the early 2000s, Poss and colleagues^4^ have performed the amputation of 20% of the ventricular cardiac apex and observed a reconstitution of a functionally perfect tissue in just 30 days without the formation of a fibrous scar in the latter years, numerous experiments were done to understand the mechanisms underlying this process. The activation of the epicardium has evidenced an implementiation of the stem-cell pool^5^, together with dedifferentiation of CMs^3,6,30^ and possibly the cardiac stem-cell residents in the myocardium^3^. The pool of regenerating CM, epicardial and endocardial cells has been collected in ex vivo ventricle culture already from 12 h post the isolation [Romano et al., personal communication]. Moreover, collecting the non-adherent cells (presumably epicardial and endocardial cells) maintained in culture supplemented with FGF, revealed 100% GATA4-positivity during 72–96 h and positivity for 90% after 15 days of culture (Romano et al., personal communication]. Recently, an involvement of miRs has been shown by the array analysis at 7 days post operation dpa^25^, and in particular of miR-133^31^. However, in the reference panorama, information is still lacking about the timing of the downregulation of the miRs, about the possible involvement of miRs-1 and of the isoforms miR-133a and -133b in the heart differentiation and regeneration. In the present study the amputation of 20% of the cardiac ventricle of adult zebrafish was done. Hearts were harvested from 24 h to 30 dpa, and analysed for miRs (miR-1 and miR-133a/miR-133b) by qPCR to know how their expression levels vary at different stages of regeneration. miR-1 is the most conserved miRNA during evolution^16^, whereas a gene duplication probably has formed the miR-133 gene, which in fact is positioned in the same genetic locus of the miR-1^31^ and, in mammals, it regulates transcription of myoD^19^.There are two members in the miR-133 family: miR-133a and miR-133b. The regulation of these miRs is a necessary event to have the transition from embryonic CMs to the functionally mature CMs in the adult^19^ [Fig. 7]. Previous studies indicated that during myogenesis, the signalling pathway of MyoD are regulated by both miR-133a and miR-133b^24^. In zebrafish, transgenic-inducing elevation of miR-133 levels after injury provoked an inhibition of myocardial regeneration, while the knockout of miR-133 showed increased CM proliferation^30^. Recently studies in the zebrafish have revealed that for a large part, the CMs and epithelial cells-from the epicardium and endocardium-are the major source of regenerating cardiac muscle and not stem–cells, as was believed^1,7,32^. The regenerating cells act in concert with the specific environment driven by FGF and other morphogenetic factors^13,21^. This finding is in line to what is observed in neonatal mice^32^ that is considered a comparable model in mammals. Similarly of the observations in mice, the zebrafish epicardium has responded to heart injuries by reactivating the expression of the embryonic genes associated under FGF stimuli (marked by raldh2 expression and presence of retinoic acid), including *tbx18, wt1b*^27,33^. Most of these genes were demonstrated to be, directly or indirectly, a target of miR-1 and miR-133a and miR-133b (ref. ^24^) [Fig. 7]. For example, among the target genes of miR-133, the genes for fibroblast growth factor receptor 1 (*FGFR1*) and protein phosphatase-2A-catalytic subunit (*PP2AC*, including *Ppp2ca* and *Ppp2cb*) seem to be promising to understand the possible induction. Both the genes participate to the signal transduction of the ERK1/2 cascade^24^. In experiments of BrdU incorporation, it was demonstrated that EPCs becomes proliferative at least from 3 days post the surgery^1,5,6^ and even before, from 24 h post the surgery^13^.

In accordance with this finding, we have explored the possibility that the activation could be already between the 1 and 2 dpa. In fact, the expression of miRNA-1, 133a and 133b during regenerative phenomenon showed a downregulation around the first 48 h post the surgery, suggesting that the transition from epicardial and other tissue has already started. In parallel with miRs analyses, the immunostaining of HSP70, WT1 and cardiac troponin (T factor) was evidenced, because they are markers of activation and myocardial cells differentiation and linked directly or indirectly to the miRs analysed. HSP70 is expressed in mammals as well as in zebrafish and acts in the folding/transport of newly synthesized proteins^34,35^. It is involved in ontogenesis, cellular metabolism in regeneration and also in blocking apoptosis^35–39^. In mice, miRs-133 (a, b) are acting as antiapoptotic signals because of directly repressing the translation of caspases, and thus they act synergistically with HSP70^40,41^. However, miR-1 is a direct repressor of HSP70 and its upregulation was demonstrated during the regeneration of zebrafish heart^5^. Some years ago, we optimized the immunohistochemistry for HSP70 antibody^42^, and recently, also for the zebrafish heart. In our finding, already after 24–48 h, a strong positivity to HSP70 in the RC has been evident. In our findings, the downregulation of miRs-133a and b started at 24 h and peaked at 2–3 days post the surgery, whereas miR-1 peaked already at 24 h. Thus, we suggest that the early expression of HSP70 is necessary to contrast the caspase protein translation in all injured hearts (due to miR-133 downregulation). The *wt1* gene encodes a zinc-finger transcription factor and RNA-binding protein that directs the EMT of EPCs by activating genes such as *vegf*, *α4integrin* and *raldh2*^43^. Thus, WT1 is considered a specific marker of heart EPCs in fish during regeneration and is linked with FGF transduction pathway^13,26,44^. The translation of *vegf* and *raldh2* are directly repressed by miR-1 (refs. ^5,13^). In mice, the epicardium bordering the ischaemic area was found to transiently re-express embryonic EPC marker genes including *wt1* and initiate proliferation^8,45,46^. In coherence to our data, WT1 + EPCs were detected early in regeneration, already after 1–2 dpa. Our observation at 2 dpa identified many extracellular matrix fibres and WT1^+^-EPCs that creep within the clot. The N component of the cardiac troponin T (cTnT) is a constituent of the contractile structures and is involved in the assembly of sarcomeres during cardiac development in zebrafish^47^. In the regenerating site from 7 days, a high number TnT + cells with myoblast shape has been observed. Despite the regenerating region not containing well-organized muscle fibres, this component of the contractile system has seemed to be already expressed in the cells from 3 dpa, as in developing heart^29^. Myofibroblasts could be differentiated from the resident differentiated fibroblasts in the heart, in case of injury in mammals^48^. These cells have contractile ability and are positive for almost all the molecules that form the sarcomeres, including troponin T^49^. In the myocardium formation, Tbx can activate the transcription factors and morphogens such as MyoD (maintained by miR-1 expression) and Myf5^50^. Tbx18, a target of miR-133a, is specific for epicardium and it would be a key transcription factor to induce the mesenchymal transient cells to differentiate into the precursors of CM^24,29^. The downregulation of miRs-133a could allow the different cascades that produce all the myo components, including the TnT expression. In endothelial cells, and thus also in endocardial cells, it was recently proven that the miR-133b is directly responsible for the repression of the connective tissue growth factor (CTGF) translation^51^. CTGF is a protein of the CCN family of extracellular matrix-associated heparin-binding proteins, involved in controlling the response to injured tissue, inflammation and angiogenesis restoration^52^.

In conclusion, in this study, the downregulation of miRs has been evidenced already after 24 hpa, thus several days before what was observed from other studies^25,30^. Particularly, miR-1, miR-133a and miR-133b have been detected in 1 dpa in different data sets of down-regulated transcripts. These data, along with the localization of epicardial precursors and differentiating CMs might suggest that the time between the first and third dpa is critical for the activation of the regenerative process. It is probable that the block of myogenic or hyperplastic role of miR-1 is crucial in activating the regeneration process. Moreover, miR-133a is probably a key miR that can activate the epicardium because it showed a more significant downregulation already at 1 dpa. Again, the miR-133b could be a key miR because of its direct control on the CTGF protein, necessary to regulate the transition in endocardial cells from epithelial to mesenchymal elements. Although different patterns of miR expression are found by transcriptome analyses during regeneration, miR-1 and miRs-133a/b seem to be commonly expressed in mammals as well as in zebrafish^53^. This study provides key clues for the experimental early activation of pro-regenerative responses in the heart of the zebrafish, and provides crucial insights for the development of therapies targeting heart disease.

## Materials and methods

### Surgery resection

The experiments were performed according to the protocol approved by the Institutional Animal Care and Use Committee (IACUC) at the University of Pittsburg. The adult zebrafish of 7.5–10 months (*N* = 72) were anaesthetized for 3 min with 0.168 mg/L of Tricaine (MS222, Sigma) and placed dorsally in a damp sponge sterile, with the ventral side facing high. The surgery was performed following the protocol developed by Poss et al.^4^. In synthesis, the ventral area below the gills at the location of the heart was engraved with a scalpel of 0.15 mm (Fine Science Tools Inc. USA). The pericardial sac was isolated and drilled to expose the ventricle, creating a slight abdominal pressure. At this point approximately 20% of ventricular apex was removed with iridectomy scissors (Fine Science Tools Inc., USA). With this protocol, 90% of the fish survived the surgery. The fish was released into the water and stimulated breathing through air bubbles created by an air pump. After 1, 2, 3, 7, 14 and 30 dpa, the fish were lethally anaesthetized with Tricaine and the regenerating hearts were collected. *N* = 9 fish per time point were considered, as well as the control samples not treated. The hearts were explanted under sterile hood with the help of an optical microscope (Zeiss). After washing them in osmolar L15 buffer (Liebovitz, Sigma), the hearts were divided in groups for the analysis: *N* = 3 for each time point were used for histological and immunohistochemical assay; *N* = 6 for each time point for RNA extraction and subsequent qPCR; and *N* = 6 for laser dissectomy for each group (control, 24 and 48 h).

### Total RNA extraction, qPCR in whole heart (qRT-PCR) and analysis

#### Total RNA extraction

The hearts were homogenized with 1 ml Trizol (Invitrogen) for 50 mg of tissue. Then were added 0.2 ml chloroform per each ml of the used Trizol. The samples have been put in shaking for 15 s, left in ice and centrifuged with 13,000 rpm for 20 min at 4 °C. The supernatant with RNA was transferred in a new eppendorf. RNA was then precipitated with 0.5 ml of isopropanol (2-isopropanol, Sigma) per each ml of Trizol used. The samples were incubated for a night at −20 °C and, after that, centrifuged at 13,000 rpm for 20 min at 4 °C; the new pellet was re-suspended in 1 ml ethanol/75% DEPC and centrifuged. The pellet was than dried at room temperature before being re-suspended in water with diethyl-pyrocarbonate (DEPC) and stored at −80 °C. Total RNA was analysed by Pico-drop (Perkin Elmer), electrophoresis and used for RT-PCR and qRT-PCR to evaluate the relative expression of cardiac miRs.

#### Quantitative evaluation of zebrafish miRNAs in the whole hearts in different regenerative stages

The level of miRNAs was measured by qRT-PCR Mirvana miRNA Detection Kit (Ambion, Inc.) which was conjugated with the fluorescent probe SYBR Green I (Molecular Probes, Carlsbad, CA). The amplification and display of the specific products was carried out with the detection system sequences ABI Prism 7700. As internal control, the primer U6 was used for the normalization of fluorescent signals. The cycle threshold (Ct) is set within the exponential phase of PCR. The relative expression of the genes was calculated by comparing the cycle times for each target PCR. The Ct values of each target PCR were normalized by subtracting the Ct value of U6, which is represented by the value ΔCt. The level of relative expression was calculated using the following equation: relative gene expression = 2−(ΔCt_sample−ΔCt_control).

#### Microdissection experiments and qPCR on RC and epicardium (24 h and 48 h)

Fine expression of miRNAs were performed on cells extracted by microdissection. For this reason, a different procedure, more sensitive, was performed for the RNA purification and consequent qPCR.

Fish hearts were sampled after 24 and 48 h post the amputation (1 and 2 dpa). The hearts were included in Kilik-BioOptica OCT (BioOptica, IT), frozen and transported in dry ice, from which 10 µM sections were cut to the CM1850 cryogenic microtome (Leica, GER), posed on microdisplay slides (Slice PPS membrane 1.2 μM, Leica, GER) and fixed in 75% ethanol at −2 °C, stained with Histo Gene LCM Staining Kit Arcturuse (Thermo Fisher Scientific, US) and finally dehydrated in ethyl alcohol at reduced concentration and dried under vacuum.

The microdissection of the portions of the epicardium adjacent to the regenerating portion and the inner most RC cells were done on the microdisplay slides of heart by a laser Microdissector (LMD 6, Leica,GER). The microdissected parts were collected in separate 500 μL sterile and RNA-free tubes (Eppendorf, US), where extraction and purification of RNA with RNAqueous Micro Kit Ambionper (Thermo Fisher Scientific, US) was also performed to obtain miRNA. Total RNA quality-control was performed with Bioanalyzer 2100 Agilent (RNA 6000 Pico Kit Agilent Agilent, CA, USA).

Subsequently, with the use of thermocyclers: PCR mastercycler personal (Eppendorf, USA) and a real-time-PCR One-Step (Applied Biosystems, USA), we performed the procedures of miRNA retrotranscription. Consequently, by using the TaqMan-Micro-RNA-Assay template and TaqMan Universal Master Mix II (Thermo Fisher Scientific, USA), the amplification was done; the negative controls were without  nucleotides (dNTPs). The relative expression of the genes was calculated by comparing the cycle times for each target PCR. The Ct values of each target PCR were normalized by subtracting the Ct value of U6 (positive control), which is represented by the value ΔCt. The level of relative expression was calculated using the following equation: relative gene expression = 2 − (ΔCt_sample − ΔCt_control).

### Statistical analysis

For qPCR, significant differences (probably values) between the experimental group and the control/untreated fish were calculated by means of a one-tailed Student’s *t*-test from three different experiments. Data (mean ± SD) were analysed using the GraphPad Prism 3.0 Software. The level for accepted statistical significance was *P* < 0.001.

### Histology and immunohistochemistry

The control and the treated samples to stages 2, 3, 7, 14 and 30 dpa were fixed in 4% paraformaldheide  (PFA) at 4 °C overnight. Subsequently, the samples were washed twice in phosphate-buffered saline (PBS) and then were dehydrated in 100% methanol. The samples were then transferred to 100% methanol in a freezer at −20 °C for storage. For use, the samples were transferred from the pure methanol solutions to a mix of methanol/ethanol at increasing proportions of the latter (2:1, 1:1, 1:2). Subsequently, they were transferred to pure toluene and finally embedded in paraffin. Histological and immunohistochemical reactions were performed by including the heart's cut sections of 7 µM using a microtome Reichert-OME; these were then mounted on glass slides treated with APES or coated with poly-l-lysine (Sigma-Aldrich).

#### Masson’s trichrome staining

The deparaffinized sections were treated with Bouin’solution (picric acid/saturated formalin/acetic acid) prewarmed to 56 °C for 15 min. The slides were cooled in running tap water and stained with Weiger ferric haematoxylin (Sigma-Aldrich) for 5 min; subsequently they were washed in water and treated with a solution of Ecarlate Biebrich-Acid Fuchsin (Sigma-Aldrich). The slides were again washed in water and then placed in the solution of F/F (phosphotungstic acid/phosphomolybdic acid) (Sigma-Aldrich) and stained in aniline blue (Sigma-Aldrich). After a rinse in 1% acetic acid, they were rinsed in deionized water, dehydrated in alcohol, cleared in toluene and mounted with Entellan.

#### Immunohistochemistry

The sections were deparaffinized and rehydrated, and then be placed in a citrate solution with 1% Triton X-100 at pH 6 and they were boiled in a pressure cooker. Subsequently, the slides were transferred to PBSTX 1% (PBS/Tween 20/Triton X-100, pH 7.3) and then in PBSTX 0.8% (pH 7.3) for 10 min. Slides were then treated with PBST (pH 7.3) + H_2_O_2_ in agitation. The sections are treated with Acetone (100%) at −20 °C and then in a block-positive solution (PBSTX 0.1% + 0.1% DMSO) for 30 min in agitation. At the end, the primary antibody was added at a concentration of 1:100 for HSP70 (BR222, Sigma-Aldrich, USA], 1:50 for WT1 or 1:300 for cTnT (cardiac Troponin T; Abcam, UK), diluted in the solution PBSTX 0.8%/normal horse serum (NHS) and 1% for HSP70 and cTnT, or PBSTX 0.8%/normal rabbit serum (NRS) and 1% for WT1, and incubated overnight at room temperature. The next day, the slides were subjected to successive washes in PBSTX 0.8% and placed in a solution of PBSTX 0.8%/NHS 1/2% BSA (or PBSTX 0.8%/NRS and1/2% BSA) for 20 min; were subsequently incubated for 1 h with the secondary antibody i.e., horse anti mouse diluted 1:800 in the solution of PBSTX 0.8% + BSA 5% for HSP70 and cTnT, or with goat anti rabbit diluted 1:2000 in the same solution for WT1. After subsequent washings in PBSTX 0.8%, the sections were washed in Tris-HCl with salt and incubated with ABC (10 μl A + 10 μl B in 2 ml of Tris-HCl with salt) for 30 min. Following treatment was washed in Tris-HCl with salt. The reaction was unmasked with DAB (3-3′-diaminobenzidine) + nickel ammonium sulfate in Tris-HCl (W/0 NaCl) + H_2_O_2_. Subsequently, the slides were washed in distilled water and PBSTX 0.8% before being dehydrated and mounted with Entellan. The sections were finally observed with microscope Axioscope (Zeiss) supported programs with image analysis (KS300, Zeiss).

## Electronic supplementary material

supplemental material

## References

[1] Kikuchi K (2014). Advances in understanding the mechanism of zebrafish heart regeneration. Stem Cell Res.

[2] Chimenti I (2010). Cardiosphere-derived cells transplanted into infarcted mice relative roles of direct regeneration versus paracrine effects of human. Circ. Res..

[3] Wu C, Weidinger G (2014). Zebrafish as a model for studying cardiac regeneration. Curr. Pathobiol. Rep..

[4] Poss KD, Wilson LG, Keating MT (2002). Heart regeneration in zebrafish. Science.

[5] Lepilina A (2006). A dynamic epicardial injury response supports progenitor cell activity during zebrafish heart regeneration. Cell.

[6] González-Rosa JM, Peralta M, Mercader N (2012). Pan-epicardial lineage tracing reveals that epicardium derived cells give rise to myofibroblasts and perivascular cells during zebrafish heart regeneration. Dev. Biol..

[7] Jopling C (2010). Zebrafish heart regeneration occurs by cardiomyocyte dedifferentiation and proliferation. Nature.

[8] van Wijk B, Gunst QD, Moorman AFM, van den Hoff MJB (2012). Cardiac regeneration from activated epicardium. PLoS ONE..

[9] Munoz-Chapuli R (2002). The epicardium and epicardial-derived cells: multiple functions in cardiac development. Rev. Exp. Cardiol..

[10] Ieda M (2009). Cardiac fibroblasts regulate myocardial proliferation through beta1 integrin signaling. Dev. Cell.

[11] Ausoni S, Sartore S (2009). From fish to amphibians to mammals: in search of novel strategies to optimize cardiac regeneration. J. Cell Biol..

[12] Hall CL, Wang C, Lange LA, Turley EA (1994). Hyaluronan and the hyaluronan receptor RHAMM promote focal adhesion turnover and transient tyrosine kinase activity. J. Cell Biol..

[13] Missinato MA, Tobita K, Romano N, Carroll JA, Tsang M (2015). Extracellular component hyaluronan and its receptor Hmmr are required for epicardial EMT during heart regeneration. Cardiovasc. Res..

[14] Jakob P, Landmesser U (2012). Role of microRNAs in stem/progenitor cells and cardiovascular repair. Cardiovasc. Res..

[15] Zhao Y, Srivastana D (2007). A developmental view of microRNA function. Trends Biochem. Sci..

[16] Kloosterman WP (2006). Cloning and expression of new microRNAs from zebrafish. Nucl. Acid Res..

[17] Sayed D, Abdellatif M (2011). MicroRNAs in development and disease. Physiol. Rev..

[18] Seeger FH, Zeiher AM, Dimmeler S (2013). MicroRNAs in stem cell function and regenerative therapy of the heart. Arterioscler. Thromb. Vasc. Biol..

[19] Gama-Carvalho M, Andrade J, Brás-Rosário L (2014). Regulation of cardiac cell fate by microRNAs: implications for heart regeneration. Cell.

[20] van Rooij E (2007). Control of stress-dependent cardiac growth and gene expression by a microRNA. Science.

[21] Wang J, Karra R, Dickson AL, Poss KD (2013). Fibronectin is deposited by injury-activated epicardial cells and is necessary for zebrafish heart regeneration. Dev. Biol..

[22] Liu N (2008). MicroRNA-133a regulates cardiomyocyte proliferation and suppresses smooth muscle gene expression in the heart. Genes Dev..

[23] Torella D (2011). MicroRNA-133 controls vascular smooth muscle cell phenotypic switch in vitro and vascular remodeling in vivo. Circ. Res..

[24] Feng Y (2013). A feedback circuit between miR-133 and the ERK1/2 pathway involving an exquisite mechanism for regulating myoblast proliferation and differentiation. Cell Death Dis..

[25] Yin VP, Lepilina A, Smith A, Poss KD (2012). Regulation of zebrafish heart regeneration by miR-133. Dev. Biol..

[26] Serluca F (2008). Development of the proepicardial organ in the zebrafish. Dev. Biol..

[27] Bollig F (2006). Identification and comparative expression analysis of a second*wt1* gene in zebrafish. Dev. Dyn..

[28] Braitsch CM, Yutzey KE (2013). Trascriptional control of cell lineage development in epicardium-derived cells. J. Dev. Biol..

[29] Huang W, Zhang R, Xu X (2009). Myofibrillogenesis in the developing zebrafish heart: a functional study of tnnt2. Dev. Biol..

[30] Lien C, Harrison MR, Tuan T, Starnes VA (2012). Heart repair and regeneration: recent insights from zebrafish studies. Wound Repair Regen..

[31] Viravuth PY, Lepilina A, Smith A, Poss KD (2012). Regulation of zebrafish heart regeneration by miR-133. Dev. Biol..

[32] Beltrami AP, Cesselli D, Beltrami CA (2012). Stem cell senescence and regenerative paradigms. Clin. Pharmacol. Ther..

[33] Cai CL (2008). A myocardial lineage derives from Tbx18 epicardial cells. Nature.

[34] Krone PH, Sass JB, Lele Z (1997). Heat shock protein gene expression during embryonic development of the zebrafish. Cell Mol. Life Sci..

[35] Evans TG, Yamamoto Y, Jeffery WR, Krone PH (2005). Zebrafish HSP70 is required for embryonic lens formation. Cell Stress Chaperones.

[36] Senf SM, Howard TM, Ahn B, Ferreira LF, Judge AR (2013). Loss of the inducible HSP70 delays the inflammatory response to skeletal muscle injury and severely impairs muscle regeneration. PLoS ONE.

[37] Beere HM (2005). Death versus survival: functional interaction between the apoptotic and stress- inducible heat shock protein pathways. J. Clin. Invest..

[38] Benjamin IJ, McMillan DR (1998). Stress (heat shock) proteins: molecular chaperones in cardiovascular biology and disease. Circ. Res..

[39] Bruns AF (2012). A heat-shock protein axis regulates VEGFR2 proteolysis, blood vessel development and repair. PLoS ONE.

[40] Xu C (2007). The muscle-specific microRNAs miR-1 and miR-133 produce opposing effects on apoptosis by targeting HSP60, HSP70 and caspase-9 in cardiomyocytes. J. Cell Sci..

[41] Jovanovic M, Hengartner MO (2006). MiRnas and apoptosis: RNAs to die for. Oncogene.

[42] Mosca F (2013). Heat shock protein 70 KDa (HSP70) increase in sea bass (*Dicentrarchus labrax* L.,1758) thymus after vaccination against *Listonella anguillarum*. Fish Physiol. Biochem..

[43] Schnabel K, Wu CC, Kurth T, Weidinger G (2011). Regeneration of cryoinjury induced necrotic heart lesions in zebrafish is associated with epicardial activation and cardiomyocyte proliferation. PLoS ONE.

[44] Scholz H, Kirschner M (2011). Oxygen-dependent expression in development and in cancer: lessons learned from the Wilms’tumor gene WT1. Front. Mol. Neurosci..

[45] Zhou B (2011). Adult mouse epicardium modulates myocardial injury by secreting paracrine factors. J. Clin. Invest..

[46] von Gise A (2011). WT1 regulates epicardial epithelial to mesenchymal transition through β-catenin and retinoic acid signaling pathways. Dev. Biol..

[47] Sehnert AJ (2002). Cardiac troponin T is essential in sarcomere assembly and cardiac contractility. Nat. Genet..

[48] Gabbiani G, Ryan GB, Majno G (1971). Presence of modified fibroblasts in granulation tissue and their possible role in wound contraction. Experientia.

[49] Ahmad F (2008). The role of cardiac troponin T quantity and function in cardiac development and dilated cardiomyopathy. PLoS ONE.

[50] Fujii T, Tsunesumi S, Yamaguchi K, Watanabe S, Furukawa Y (2011). Smyd3 is required for the development of cardiac and skeletal muscle in zebrafish. PLoS ONE.

[51] Chen CC, Lau LF (2009). Functions and mechanisms of action of CCN matricellular proteins. Int. J. Biochem. Cell Biol..

[52] Yang L, Hou J, Cui XH, Suo LN, Lv YW (2017). MiR-133b regulates the expression of CTGF in epithelial-mesenchymal transition of ovarian cancer. Eur. Rev. Med. Pharmacol. Sci..

[53] Crippa S (2016). Comparative transcriptome profiling of the injured zebrafish and mouse hearts identifies miRNA-dependent repair pathways. Cardiovasc. Res..

