# Supplementary figures and images for: Micro RNAs are involved in activation of epicardium during zebrafish heart regeneration
Source: Cell Death Discov. 2018 Mar 12;4:41. doi: 10.1038/s41420-018-0041-x (PMC5849881; doi:10.1038/s41420-018-0041-x)

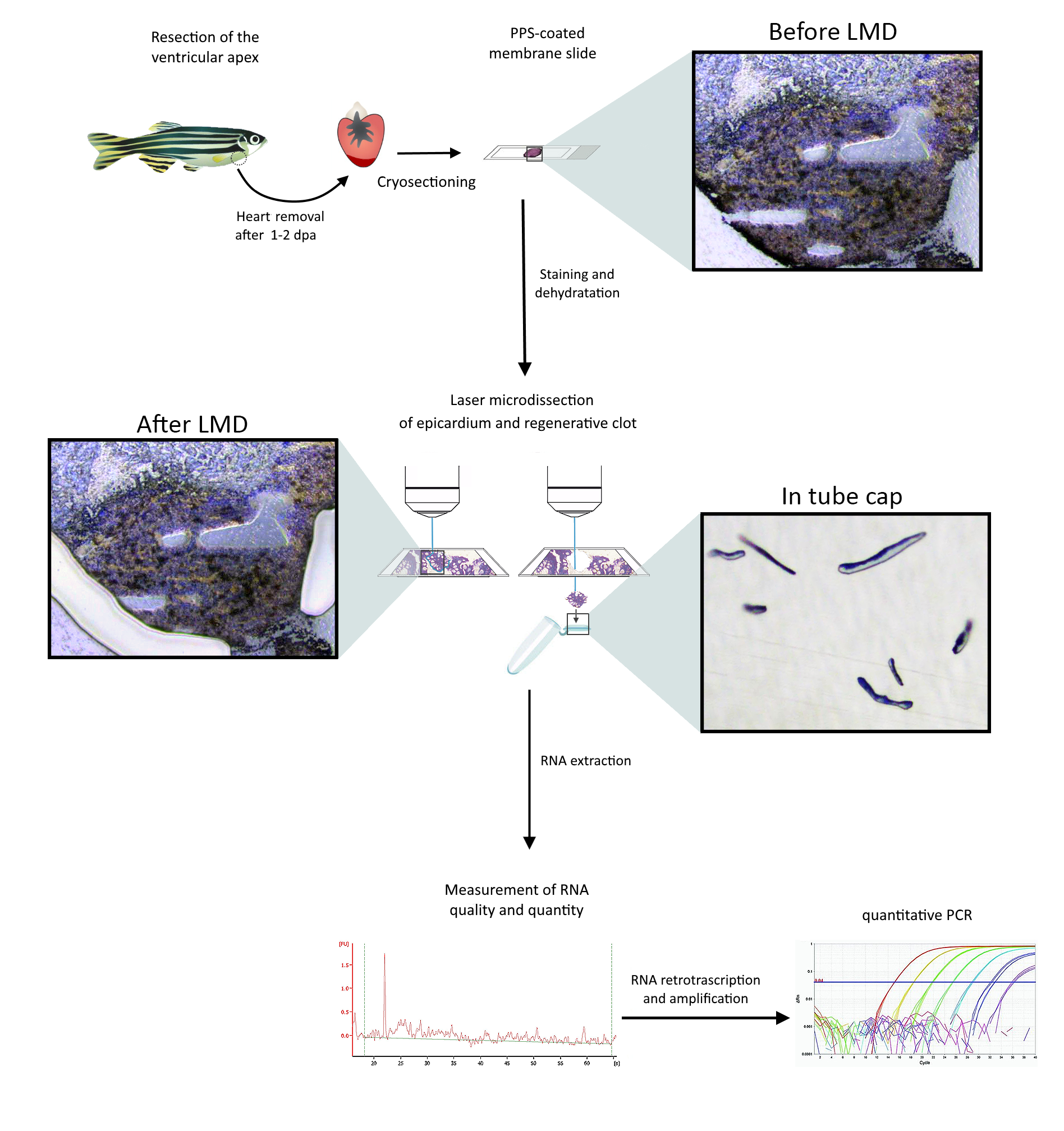

Supplement: Supplementary file 1 — supplemental material [file 41420_2018_41_MOESM1_ESM.bmp]
